# Comparison of oral health behaviour between dental and non-dental undergraduates in a university in southwestern China——exploring the future priority for oral health education

**DOI:** 10.1186/s12903-020-01232-1

**Published:** 2020-09-07

**Authors:** Mingming Li, Zhiwu Wu, Rui Zhang, Lei Lei, Siqi Ye, Ran Cheng, Tao Hu

**Affiliations:** 1 Department of Preventive Dentistry, State Key Laboratory of Oral Diseases, West China Hospital of Stomatology, Sichuan University, Chengdu, Sichuan China; 2 West China school of Stomatology, Sichuan University, Chengdu, Sichuan China

**Keywords:** Oral health education, Oral health behaviour, Dental students, Non-dental students

## Abstract

**Background:**

Based on a national survey in 2015, people’s oral health behaviour (OHB) has not kept up with the pace of knowledge and attitudes in China after decades of oral health education (OHE). Thus, we need to improve OHE to strengthen people’s OHB. Undergraduates are regarded as the best candidates for the improvement of OHE. The objective of this study is to determine undergraduates’ oral health status and existing problems in OHB by comparing dental and non-dental students at Sichuan University. We hope to provide some suggestions for future OHE to improve people’s OHB.

**Methods:**

A quasi-experimental study designed with a pre-test and post-test group was conducted. A total of 217 dental students and 135 non-dental students were enrolled. They were administered an OHE course focused on OHB. A survey about oral health behaviour and knowledge was conducted before and after the course.

**Results:**

According to the pre-course survey, dental students surpassed non-dental students in terms of toothbrushing frequency, method, and time, but unfortunately, flossing was overlooked by all the students. After the course, both dental and non-dental students showed strong willingness to improve their OHB. More non-dental students than dental students were willing to use toothpicks and Chinese herbal toothpaste before and after the course.

**Conclusions:**

OHE focused on behaviour has a positive effect on university students. Future OHE and interventions should focus on flossing, toothbrushing methods, toothpicks, Chinese herbal toothpaste and modifications to adopt new media.

## Background

Oral disease is a worldwide epidemic and has imposed an enormous burden on the health and economy of the whole society [1]. The number of people with untreated oral conditions worldwide increased from 2.5 million in 1990 to 3.5 billion in 2015, with a 64.0% increase in disability-adjusted life years due to oral conditions [2]. Among these conditions, untreated dental caries, severe periodontitis, and missing teeth are the three most common and chronic infectious oral diseases [1].

Fortunately, most oral diseases, especially dental caries and periodontal diseases, are largely preventable through various promotion interventions. Oral health education (OHE) was once considered the most cost-effective intervention [3]. In China, OHE has been conducted for years. The national campaign, “National Teeth Love Day”, was established in 1989 and has proposed a topic for OHE every year [4]. After decades of effort, the national survey in 2015 showed that approximately 60.0% of citizens had basic knowledge regarding oral health, and 84.9% of them had a positive attitude [5]. However, the caries rates of children aged 3–5 years and elderly people aged 65–74 years were 62.5 and 98.0%, respectively, which were much higher than the rates 10 years ago. In addition, 87.4% of adults between 35 and 44 years old suffered from gingival bleeding [5]. These findings may suggest that people’s oral health behaviour (OHB) has not improved with the pace of the knowledge and attitudes in China via OHE.

Some recent studies have indicated that among various interventions, motivational interviewing (MI) interventions and counselling interventions are effective in promoting OHB, while OHE is effective for knowledge and attitude but ineffective, or have only a short-term effect, for behaviour [6, 7]. Some reviews have suggested that the psychology of behaviour change is the key to oral health promotion. MI is currently one of the most effective forms of psychological intervention [8]. However, some studies also suggest that the application of MI in dental healthcare shows a null effect or remains controversial [9, 10]. More importantly, MI was developed for individual promotion in a clinical setting. It is worth noting that individual prevention may not be enough to achieve sustainable improvements at the population level [11]. In China, due to its large population, the lack of dentists, dental hygienists and medical resources (the dentist-to-population ratio is 1:10,000 in China [12]), MI may not be the most cost-effective method. Instead, OHE is more suitable. A recent study has suggested that the educational interventions carried out by health professionals still have the potential to promote OHB within the population [13]. Computer-aided, video-assisted, text-message-assisted and quantitative light-induced fluorescence technology-based learning in OHE has recently been shown to have positive impacts on behaviour [14–17]. Thus, based on our current situation, we still have room to improve OHE to strengthen people’s oral health, especially OHB.

Since 2016, several oral-related policies have been enacted to improve OHE in China. For example, OHE should be improved in preschool and primary school [18]. However, children are often too young to accept OHE and fail to do well in OHB. Their oral health mainly depends on their parents’ correct guidance over a long period of time [19]. Many surveys have revealed that parents’ behaviours are associated with children’s OHB [20–22]. China is also facing an ageing and undereducated population and an upcoming socio-economic burden resulting from the elderly population [23, 24]. Several reports have shown that oral health is positively correlated with education [25, 26]. The offspring of elderly people may contribute to improving the health of their parents by transferring knowledge and practices [27–29]. As a result, undergraduates might be the best candidates for OHE. First, they are well educated and more receptive to OHE. It has been verified that OHE positively changes the behaviour of college students [30]. Second, younger adults can act as OHE assistants for their parents. In-school undergraduates aged between 18 and 22 years are at an important phase of transition from adolescence to adulthood [31]. They will become parents in a few years and be beneficial to their future children. The earlier OHB is established, the more benefits it will bring about, and the longer it will be retained [30]. Third, it is comparably easy to arrange OHE in school.

However, according to the WHO guidelines, the undergraduate group was not included in the Chinese national survey. Their oral health status was unknown for years. It is necessary to know the current status of their oral health and the existing problems concerning OHB. In this survey, the differences in oral health behaviour and knowledge between dental and non-dental students at Sichuan University were compared to identify the current problems in OHB and to provide advice for better OHE.

## Methods

### Participants

Third-year undergraduate dental students (in the first year of their professional dental education) were enrolled. The inclusion criteria were a) third-year dental students at Sichuan University and b) agreement to participate in the survey. The exclusion criterion was incorrectly written answers.

Second- to fourth-year non-dental students were also enrolled. The inclusion criteria were a) students at Sichuan University and b) agreement to participate in the survey. The exclusion criteria were a) dental students and b) incorrectly written answers.

A pre-test was conducted on the toothbrushing frequency of dental and non-dental students. In order to estimate the sample size, the method “difference test of rate comparison between two groups” was chose and the calculator (1 + 1/*k*)(*μ*_*α*_ + *μ*_*β*_)^2^*p*(1 − *p*)/*δ*^2^ was applied [32] ($p=\frac{p1+ kp2}{1+k},p1=0.97,p2=0.86$). The sample size was estimated to be more than 110 in each group.

### Design

A quasi-experimental survey with a pre-test and a post-test group was conducted for the study. (The survey questionnaires are supplemented as additional files at the end of the article).

### Intervention and instruments

The dental and non-dental students received the pre-course survey on knowledge and behaviour of oral health before the lecture. The OHE was scheduled in a 90-min course by the same teacher. The content was designed based on the textbook “Preventive Dentistry” [2] and focused on the aetiology of common oral diseases and specific oral hygiene measures with the aim of improving students’ OHB. Non-dental students had the same course in the part of “behavioural guidance” as dental students. A post-course survey including the same items was conducted.

### Data collection and analysis

All the students finished the questionnaires by cell phone or by computer through “Questionnaire Star”, a professional online survey, measurement and voting platform. For the scale items in the questionnaire, we used SPSS 16.0 (IBM Corp. New York, NY, USA) to analyse the Cronbach’s alpha coefficient. Six experts (1 professor, 3 associate professors and 2 lecturers) evaluated the content validity [33], clarity, and conciseness of the questionnaires. The data are presented as percentages, means and standard deviations (SDs). The Wilcoxon signed-rank test, chi-square test and Fisher’s exact test were used for statistical analysis with SPSS 16.0. *P* < 0.05 was regarded as a statistically significant difference.

## Results

**Fig. 1:**
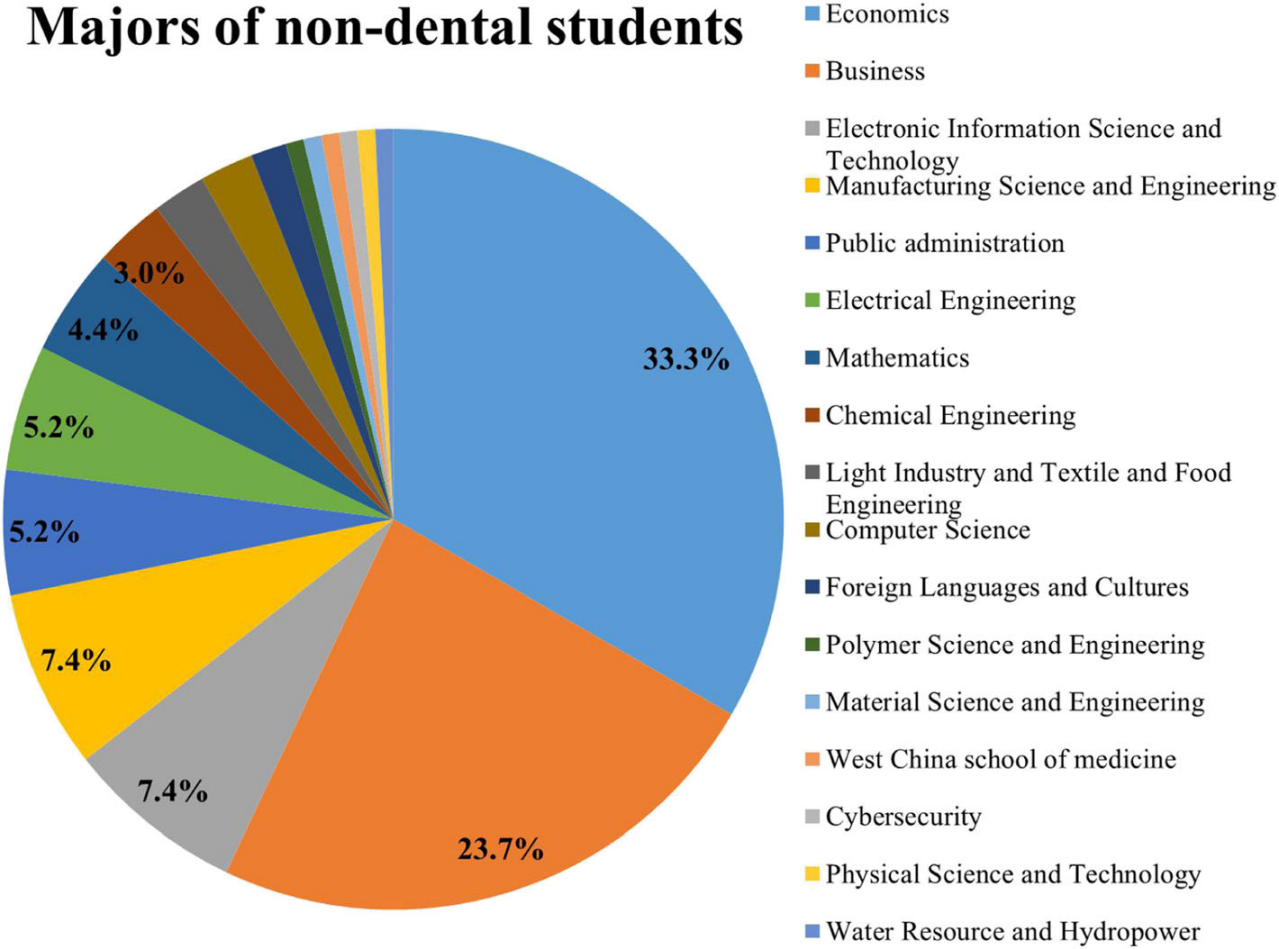
Majors of the non-dental students. The top four were the Department of Economics, Business, Electronic Information Science and Technology and Manufacturing Science and Engineering

In the pre-course survey, 217 third-year undergraduate dental students (86 male, 131 female; aged 21.3 ± 1.0 years) and 135 non-dental students (55 male, 80 female; aged 21.4 ± 0.8 years) were enrolled. In the post-course survey, 1 student in the non-dental group was excluded as a result of incorrect writing. The non-dental students were in the second to fourth years. They were from 17 departments of Sichuan University, and their majors are displayed in Fig. 1.

Reliability analysis of the scale items of a simultaneous survey showed that the Cronbach’s alpha coefficient was 0.781 for the pre-course survey and 0.711 for the post-course survey. For non-scale items in this survey, the reliability was considered acceptable when the same group completed the questionnaires at the same time. A content validity index (CVI) was calculated for the questionnaire items. The item-level CVIs and the scale-level CVIs were 1.

**Fig. 2:**
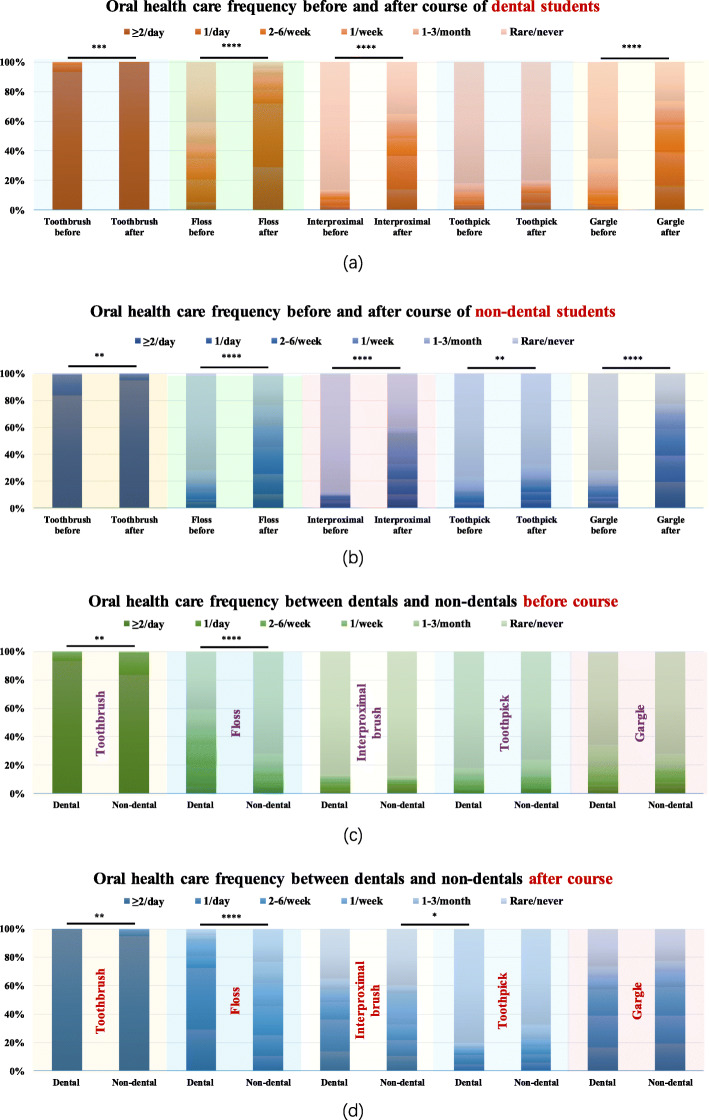
Oral health care frequency of dental students and non-dental students before and after the course. **a**, **b** Dental and non-dental students improved greatly in toothbrushing and interproximal cleaning after the OHE-related course. **c**, **d** Dental students surpassed non-dental students in toothbrushing (*P* = 0.004) and flossing (*P* = 0.000) before and after the course. Non-dental students were more willing to use toothpicks after the course (*P* = 0.016). (Wilcoxon signed-rank test; ****, *P* = 0.000; **, *P* = 0.01; *, *P* < 0.05)

First, we surveyed the oral health care frequency of dental and non-dental students (Fig. 2). Before the course (Fig. 2c), most students in both groups did well at brushing their teeth twice a day. Up to 71.9% of students in the non-dental group and 40.6% in the dental group never used floss (Fig. 2c). However, the dental group was still significantly better than the non-dental group (*P* = 0.000). There was no obvious difference between the two groups in the use of interproximal brushes or toothpicks (*P* > 0.05). After the course (Fig. 2d), students tended to brush their teeth twice a day, with an obvious increase in both groups (*P* = 0.001 in the dental group, *P* = 0.004 in the non-dental group). The dental group was more willing to use dental floss or to use it more often (*P* = 0.000). However, one-fifth of the non-dental students were still reluctant to floss. Compared to dental students, non-dental students were more willing to use toothpicks (*P* = 0.016). In short, the dental group performed better than the non-dental group in toothbrushing and flossing before and after the course. Table 1 shows students’ knowledge about water/air flossing. Before the course, both groups were unfamiliar with these tools. The course introduced new flossing equipment to them.

**Table 1:** The knowledge about water/air flossing

	Dental students	Non-dental students	*P*^a^
Before lecture	2.83 ± 1.17	2.51 ± 1.05	0.000
After lecture	4.51 ± 0.51	4.31 ± 0.55	0.000
*P* ^b^	0.000	0.000	

Wilcoxon signed-rank test; Scale: 1 (strongly unknown) to 5 (strongly knowledgeable)]

^a^ Comparison between dental and non-dental students before and after class

^b^ Comparison between students before and after class

**Fig. 3:**
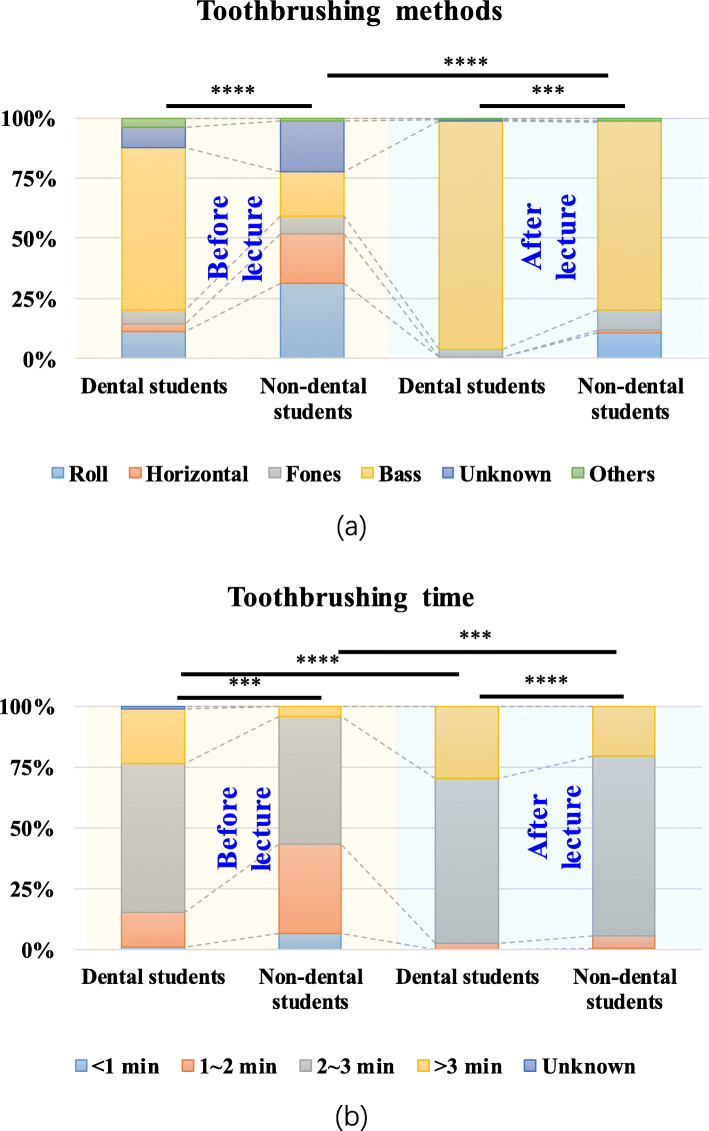
The choice of toothbrushing methods and time. **a** Dental students overwhelmed non-dental students in the use of the Bass method (*P* = 0.000). A total of 20.7% of non-dental students were using the wrong horizontal method. **b** Dental students performed generally better than non-dental students in toothbrushing time before (*P* = 0.000) and after (*P* = 0.025) the course (Wilcoxon signed-rank test; ****, *P* = 0.000; ***, *P* = 0.001)

Next, we surveyed the toothbrushing method (Fig. 3a). The number of dental students using the Bass method overwhelmed that of non-dental students (*P* = 0.000). One-fifth of the non-dental students were still using the wrong horizontal method, and one-fifth did not know the in-use methods. Table 2 shows the knowledge of the Bass method between the two groups. The results revealed that dental students performed much better than non-dental students before (*P* = 0.000) and after (*P* = 0.000) the course. Regarding brushing time (Fig. 3b), dental students performed generally better than non-dental students before (*P* = 0.000) and after (*P* = 0.025) the course. The number of students who were willing to brush their teeth for more than 2 min increased in the two groups after the course (*P* = 0.001 in the dental group, *P* = 0.000 in the non-dental group).

**Table 2:** Knowledge of the modified Bass method

	Dental students	Non-dental students	*P*^a^
**Before lecture**	4.23 ± 0.75	2.83 ± 1.16	0.000
**After lecture**	4.75 ± 0.44	4.35 ± 0.49	0.000
***P*** ^**b**^	0.000	0.000	

Wilcoxon signed-rank test; Scale: 1 (strongly unknown) to 5 (strongly knowledgeable)]

^a^ Comparison between dental and non-dental students before class

^b^ Comparison between students before and after class

**Fig. 4:**
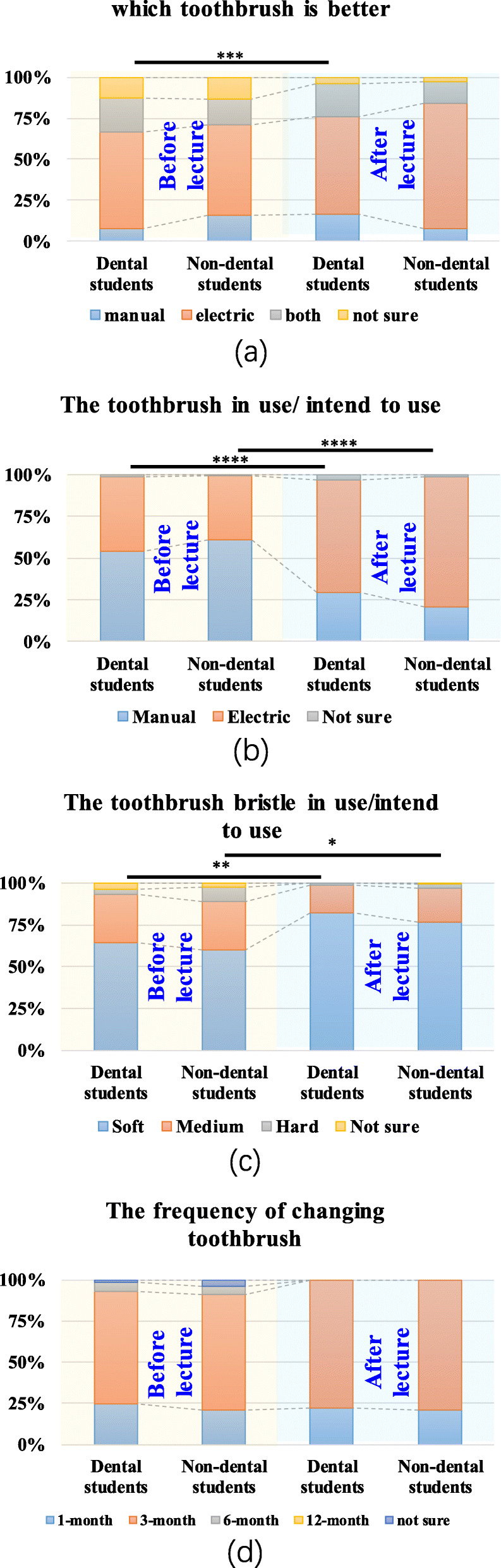
The types and replacement frequency of toothbrushes of dental students and non-dental students. **a, b** Before the course, most students in both groups preferred electric toothbrushes to manual toothbrushes. However, the actual usage of electric toothbrushes was low. After the course, more dental students thought electric toothbrushes were better. **c** Soft-bristled toothbrushes were favoured by more students after the course. **d** There was no obvious difference in the frequency of changing toothbrushes between the two groups (*P* > 0.05) (Wilcoxon signed-rank test; ****, *P* = 0.000; ***, *P* = 0.001; **, *P* = 0.01; *, *P* < 0.05)

We further investigated the types and replacement frequency of toothbrushes (Fig. 4). Before the course, more than half of the students in both groups preferred electric toothbrushes to manual toothbrushes (Fig. 4a). However, the actual usage or willingness to use was lower (Fig. 4b). After the course, more dental students thought electric toothbrushes were better and wanted to use them. With regard to the softness of bristles (Fig. 4c), soft-bristled toothbrushes were favoured after the course (*P* = 0.01 in the dental group, *P* = 0.012 in the non-dental group). Toothbrushes need to be replaced regularly, and most students had a good habit of changing their toothbrushes every 3 months (Fig. 4d). There was no obvious difference in these 2 items between the two groups (*P* > 0.05).

**Fig. 5:**
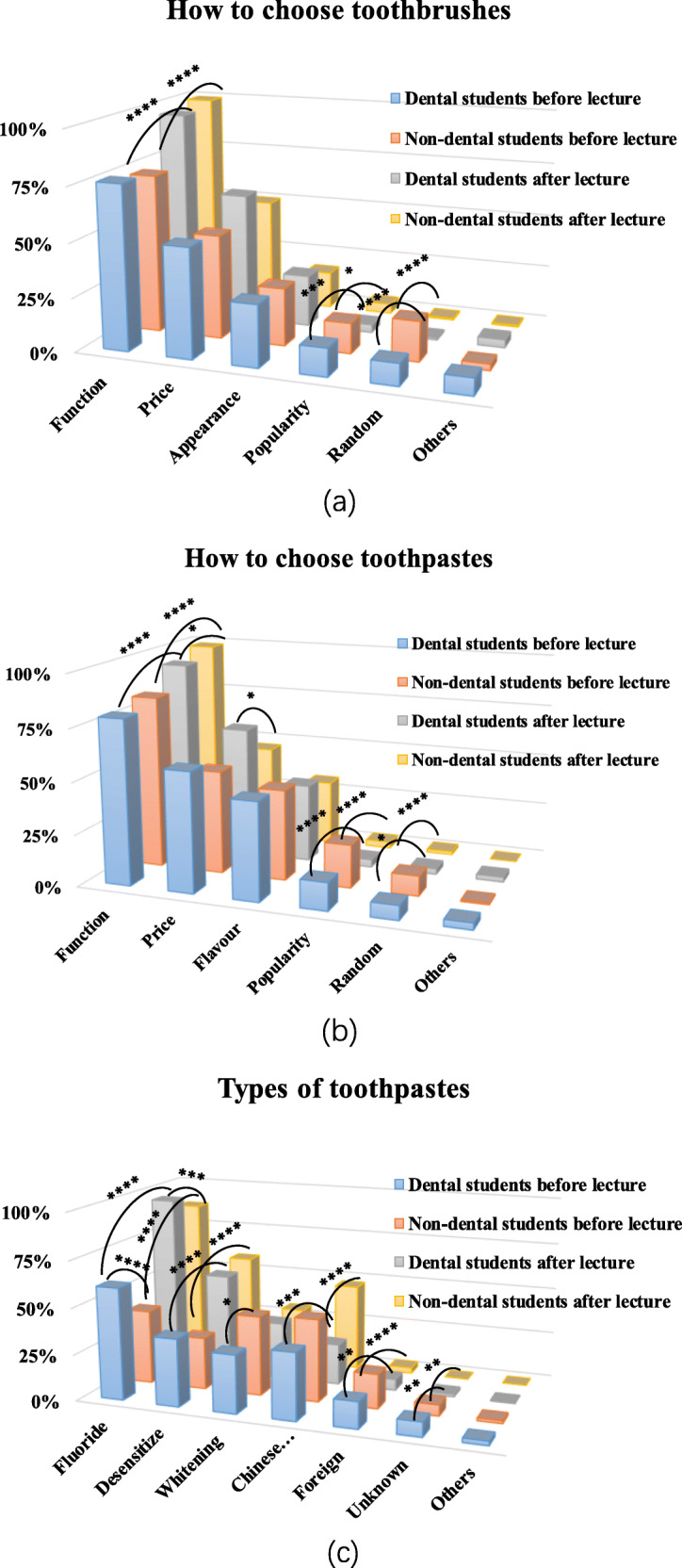
Dental students’ and non-dental students’ considerations when selecting toothbrushes and toothpaste before and after the course. **a, b** Function and price were the first two considerations. Some students cared about popularity before the course. **c** Non-dental students preferred Chinese herbs, whitening and fluoride toothpastes before the course, while dental students preferred fluoride, Chinese herbs and desensitizing toothpastes. After the course, fewer dental students and more non-dental students were willing to use Chinese herbal toothpaste (*P* = 0.000), and the two groups showed opposite trends (chi-square test or Fisher’s exact test; ****, *P* = 0.000; ***, *P* = 0.001; **, *P* = 0.01;*, *P* < 0.05)

Finally, we examined students’ considerations when selecting toothbrushes and toothpaste (Fig. 5). Before the course, function and price were the aspects of concern for most students (Fig. 5a, b). After the course, in both groups, more students realized the importance of function (*P* = 0.000), fewer students cared about popularity (*P* = 0.000) and fewer students were confused about choosing toothbrushes and toothpaste (*P* = 0.000). Regarding the function of toothpaste (Fig. 5c), non-dental students preferred Chinese herbs, whitening and fluoride toothpastes before the course, while dental students preferred fluoride, Chinese herbs and desensitizing toothpastes. After the course, more students chose fluoridated and desensitizing toothpaste in both groups (*P* = 0.000). Fewer dental students (*P* = 0.000) and more non-dental students (*P* = 0.000) were willing to use Chinese herbal toothpaste, and the two groups showed opposite trends.

## Discussion

The 4th Chinese National Oral Health Survey showed that people’s oral health knowledge and attitudes had been greatly improved, but caries and periodontal diseases remained serious problems, and people’s OHB was far from standard. OHE focused on behaviour for undergraduates, with the aim of helping them improve themselves and indirectly helping children and elderly people, might be an efficient way of addressing this issue.

A previous study showed that the oral healthcare knowledge and behaviour of dental students were better than those of non-dental students [34]. For dental students at Sichuan University, they underwent oral health education starting in their first year. Some clinical research or practice was open to them, such as the university students’ innovation and entrepreneurship training programme. Additionally, some of them had opportunities to get in touch with seniors and obtain additional information. The third year is the first year of professional dental education. Preclinical education and practice can enhance dental students’ knowledge and behaviour of oral health. For non-dental students who had similar general education backgrounds as those of dental students, the difference might come from pre-clinical oral health education. Therefore, we used the dental group as a reference to identify the differences or gaps in OHB between the two groups. Additionally, we explored the role of OHE focused on OHB in both dental and non-dental students by comparing their oral health knowledge and attitudes before and after the course. Regarding the behaviour guidance part of the course, non-dental students experienced the same content and degree of difficulty as did dental students. Although this relatively-professional education was more difficult than usual, it was helpful for non-dental students to understand aetiology-based knowledge and might also be helpful for behavioural education.

Toothbrushing frequency was well known among all the students. However, half of the non-dental students did not meet the recommended brushing time of 2 min. Additionally, non-dental students faced difficulty in selecting a tooth brushing method. The (modified) Bass method, Roll method, Fones method and horizontal method are the most widely used brushing methods [35]. A study has shown that the (modified) Bass technique is effective in controlling dental plaque and alleviating gingival inflammation [36]. The horizontal method, which could result in wedge-shaped defects, is not recommended. However, it is a common method used in China. In this survey, less than one-fifth of the non-dental students used the Bass method, but more than two-fifths of them used incorrect methods. From these results, we concluded that although non-dental students had good brushing frequency, their brushing time and actual brushing methods may not be appropriate.

Interproximal cleaning was extremely overlooked by both dental and non-dental students. It was surprising that few dental students floss daily. The findings revealed signs of ignorance concerning interproximal cleaning in China. Using floss in addition to toothbrushing may reduce gingivitis, plaque, or both, more than toothbrushing alone [37]. However, floss is comparably difficult to use, which may limit its application [38]. Moreover, toothpicks have a history of more than a thousand years in China and are deeply rooted in Chinese people’s minds [39]. Toothpicks are quite popular in China and can easily be found in restaurants and take-away cutlery. Interestingly, the tendency to use toothpicks increased among non-dental students after the course. OHE on avoiding the use of toothpicks should be strengthened to minimize the periodontal damage caused by improper use.

Function and price were the most important considerations when students chose toothbrushes. A study confirmed that electric toothbrushes were more effective than manual toothbrushes [40]. Before the course, more than half of the students in both groups thought electric toothbrushes were better than manual toothbrushes. However, the actual use rate was much lower, especially in the non-dental group. Furthermore, nationwide usage was much lower. A report showed that the penetration rate of electric toothbrushes in China was only 5%, while in some developed countries, it was more than 15% and even up to 40% [41]. Price might be a possible reason for this result. After the introduction of electric toothbrushes during the course, more students realized their advantages and intended to use them, even at a higher price. If electric toothbrushes are not popular because of their high price, then we should strengthen OHE on the use of manual toothbrushes instead of emphasizing the use of electric toothbrushes.

When choosing toothpaste, function and price were the first two considerations. Interestingly, more non-dental students than dental students were willing to use Chinese herbal toothpaste before and after the course. As a part of traditional Chinese medicine, Chinese herbal toothpaste may have some effects in alleviating gingival inflammation [42] and preventing caries [43]. Chinese have partiality for Chinese herbal toothpaste. Its correct usage should be addressed in future OHE courses; for example, when facing gingival bleeding caused by periodontitis, relying on herbal toothpaste instead of scaling may worsen the disease.

One issue that cannot be ignored is that some students cared about popularity when choosing toothpaste and toothbrush. This suggests a new method of OHE: new media. Recently, a large number of popular media platforms have emerged. WeChat, an interactive social media platform in China, has a wide range of young users and is used every day [44]. A study showed that the passive acquisition (moments, public accounts, and group chat) of health information through WeChat is an important medium for college students [45]. Taobao, a large online shopping platform, is preferred by young people and carries many traditional and emerging oral care products endorsed by celebrities, which is very attractive to young people who are starstruck and pursuing popularity. At the same time, it contains a wealth of pictures, videos and instructions for the products. In addition to traditional classes, WeChat groups, WeChat public accounts and Moments can be used for regular OHE as a reminder to floss and as an update to new knowledge that is not included in textbooks. Taobao links can provide vivid information about oral hygiene products, making OHE much more convenient and cost-effective.

Overall, the majority of students realized their shortcomings in OHB and had a strong willingness to improve. Our OHE course focused on behaviour had a positive effect on university students. Dental students had much better performance than non-dental students in terms of toothbrushing frequency, method, and time and floss use. This suggests that dental students know more details about oral healthcare. Future OHE should pay more attention to flossing, toothbrushing methods, toothpicks, Chinese herb toothpaste and modifications to adopt new media.

### Limitations

As a quasi-experimental study, the grades and sample numbers of dental and non-dental students were different. The post-course survey was conducted within a short period of time. The long-term change in students’ knowledge, attitudes and behaviours is unknown. Furthermore, our survey did not include clinical examinations.

## Conclusions

According to the pre-course survey, dental students significantly surpassed non-dental students in terms of toothbrushing frequency, method, and time, but floss was overlooked by all the students. After the course, both dental and non-dental students showed strong willingness to improve their OHB. Future OHE should focus on flossing, toothbrushing methods, toothpicks, Chinese herbal toothpaste and modifications to adopt new media.

## Supplementary information

**Additional file 1.** Pre-class Questionnaire.

**Additional file 2.** Post-class Questionnaire.

## Data Availability

The main data used to support the findings of this study is included within the article. The datasets generated and analysed during the current study are available from the corresponding author (chengran@scu.edu.cn) on reasonable request.

## Abbreviations

OHE: Oral health education
OHB: Oral health behaviour
CVI: Content validity index
