# Supplementary material for: Comparison of oral health behaviour between dental and non-dental undergraduates in a university in southwestern China——exploring the future priority for oral health education
Source: BMC Oral Health. 2020 Sep 7;20:249. doi: 10.1186/s12903-020-01232-1 (PMC7487576; doi:10.1186/s12903-020-01232-1)
Supplement: Supplementary file 1 — Additional file 1. Pre-class Questionnaire. [file 12903_2020_1232_MOESM1_ESM.pdf]

Pre-class Questionnaire

1. How often do you use the following methods to clean your teeth? (Single-choice)

	≥2 times per day	1 time per day	2-6 times per week	1 time per week	1-3 times per month	Rare/ never
Toothbrush	○	○	○	○	○	○
Floss	○	○	○	○	○	○
Interproximal toothbrush	○	○	○	○	○	○
Toothpick	○	○	○	○	○	○
Gargle	○	○	○	○	○	○

2. Do you know about water/air floss? (Single-choice)

Completely understand.

Basically understand.

Not sure.

Don't understand.

Don't understand at all.

3. What kind of tooth brushing method are you using currently? (Single-choice)

Roll method.

Horizontal method.

Fones method.

Bass method/modified Bass method.

Unknown.

Others_________________.

4. Do you know about Bass method/modified Bass method? (Single-choice)

Completely understand.

Basically understand.

Not sure.

Don't understand.

Don't understand at all.

5. How long do you brush your teeth each time? (Single-choice)

<1 minute

1-2 minutes.

2-3 minutes.

More than 3 minutes.

Unknown.

6. Which one do you think is better, manual toothbrush or electric toothbrush? (Single-choice)

Manual toothbrush.

Electric toothbrush.

Much the same.

Not sure.

7. Are you using or intend to use manual toothbrush or electric toothbrush? (Single-choice)

Manual toothbrush.

Electric toothbrush.

Not sure.

8. Which type of toothbrush bristles are you using? (Single-choice)

Hard bristles.

Medium bristles.

Soft bristles.

Not sure.

9. How often do you change your toothbrush? (Single-choice)

1 month.

3 months.

6 months.

12 months.

Not sure.

10. How do you choose toothbrush? (Multiple-choice)

Function.

Price.

Appearance.

Popularity.

Random.

Others _________________.

11. How do you choose toothpaste? (Multiple-choice)

Function.

Price.

Flavour.

Popularity.

Random.

Others_________________.

12. What kind of toothpaste have you used in the past year? (Multiple-choice)

Fluoride (resistant to tooth decay).

Desensitize (e.g. SENSODYNE, LESENING).

Whitening.

Chinese herbal toothpaste (e.g. Yunnan Baiyao toothpaste).

Foreign toothpaste (e.g. toothpaste purchased by Haitao).

Unknown.

Others_________________.
