# Supplementary material for: Comparison of oral health behaviour between dental and non-dental undergraduates in a university in southwestern China——exploring the future priority for oral health education
Source: BMC Oral Health. 2020 Sep 7;20:249. doi: 10.1186/s12903-020-01232-1 (PMC7487576; doi:10.1186/s12903-020-01232-1)
Supplement: Supplementary file 2 — Additional file 2. Post-class Questionnaire. [file 12903_2020_1232_MOESM2_ESM.pdf]

Post-class Questionnaire

1. After the course, how often will you use the following methods to clean your teeth? (Single-choice)

	≥2 times per day	1 time per day	2-6 times per week	1 time per week	1-3 times per month	Rare/ never
Toothbrush	○	○	○	○	○	○
Floss	○	○	○	○	○	○
Interproximal toothbrush	○	○	○	○	○	○
Toothpick	○	○	○	○	○	○
Gargle	○	○	○	○	○	○

2. After the course, do you know about water/air floss? (Single-choice)

Completely understand.

Basically understand.

Not sure.

Don't understand.

Don't understand at all.

3. After the course, what kind of tooth brushing method will you use? (Single-choice)

Roll method.

Horizontal method.

Fones method.

Bass method/modified Bass method.

Unknown.

Others_________________.

4. After the course, do you know about Bass method/modified Bass method? (Single-choice)

Completely understand.

Basically understand.

Not sure.

Don't understand.

Don't understand at all.

5. After the course, how long will you brush your teeth each time? (Single-choice)

<1 minute

1-2 minutes.

2-3 minutes.

More than 3 minutes.

Not sure.

6. After the course, which one do you think is better, manual toothbrush or electric toothbrush? (Single-choice)

Manual toothbrush.

Electric toothbrush.

Much the same.

Not sure.

7. After the course, which do you prefer, manual or electric toothbrush? (Single-choice)

Manual toothbrush.

Electric toothbrush.

Not sure.

8. After the course, which type of bristles do you prefer? (Single-choice)

Hard bristles.

Medium bristles.

Soft bristles.

Not sure.

9. After the course, how often will you change your toothbrush? (Single-choice)

1 month.

3 months.

6 months.

12 months.

Not sure.

10. After the course, how would you choose toothbrush in the future? (Multiple-choice)

Function.

Price.

Appearance.

Popularity.

Random.

Others _________________.

11. After the course, how would you choose toothpaste in the future? (Multiple-choice)

Function.

Price.

Flavour.

Popularity.

Random.

Others_________________.

12. After the course, which kind of toothpaste would you choose in the future? (Multiple-choice)

Fluoride (resistant to tooth decay).

Desensitize (e.g. SENSODYNE, LESENING).

Whitening.

Chinese herbal toothpaste (e.g. Yunnan Baiyao toothpaste).

Foreign toothpaste (e.g. toothpaste purchased by Haitao).

Unknown.

Others_________________.

13. How about the difficulty of the course for you? (Single-choice)

Very difficult.

Difficult.

Not sure.

Easy.

Very easy.

14. Did you master the knowledge of the course? (Single-choice)

Completely mastered.

Mastered.

Not sure.

Little.

Unknown.

15. Was this course helpful for you? (Single-choice)

Very helpful.

Helpful.

Not sure.

Not helpful.

Completely not helpful.
